# Serum amino acid profiles in patients with myasthenia gravis

**DOI:** 10.1007/s00726-023-03303-3

**Published:** 2023-07-20

**Authors:** Piotr Kośliński, Łukasz Rzepiński, Emilia Daghir-Wojtkowiak, Marcin Koba, Zdzisław Maciejek

**Affiliations:** 1 Department of Toxicology and Bromatology, Collegium Medicum in Bydgoszcz, Nicolaus Copernicus University in Toruń, Dr. A. Jurasza 2, 85-089 Bydgoszcz, Poland; 2 Department of Neurology, 10th Military Research Hospital and Polyclinic, Powstańców Warszawy 5, 85-681 Bydgoszcz, Poland; 3 Sanitas - Neurology Outpatient Clinic, Bydgoszcz, Poland; 4 International Centre for Cancer Vaccine Science, University of Gdansk, Gdansk, Poland

**Keywords:** Myasthenia gravis, Amino acids, Biomarkers, Mass spectrometry

## Abstract

Myasthenia gravis (MG) is an autoimmune disease characterized by weakness and rapid fatigue. Diagnostic methods used for myasthenia gravis are not conclusive and satisfactory, therefore it is necessary to develop reliable tools to help diagnose myasthenia gravis as early as possible. The aim of the study was to use HPLC–MS in conjunction with multivariate statistical analyses to investigate changes in the amino acid metabolic profiles between myasthenia gravis patients compared and controls. In addition, the effect of treatment regimens and myasthenia gravis type, on the observed changes in amino acid metabolic profiles were assessed. Serum levels of 29 amino acids were determined in 2 groups of individuals—28 patients with myasthenia gravis and 53 control subjects (CS). The results of our study indicate that serum levels of several amino acids in patients with myasthenia gravis changed significantly compared to the control group. Statistical analysis revealed differences between amino acids concentration in patients with different therapeutic scheme. In conclusion, amino acids may be involved in mechanisms underlying myasthenia gravis pathogenesis as well as may be potential biomarkers in MG patients diagnosis. However, considering the multifactorial, heterogenous and complex nature of this disease, validation on a larger study sample in further research is required before application into diagnostic practice.

## Introduction

Myasthenia gravis (MG) is an autoimmune disease that leads to dysfunction of the neuromuscular junction (Dresser et al. 2021). The developmental grounds of myasthenia gravis is related to class II hypersensitivity reaction, where IgG autoantibodies react with intracellular or extracellular antigens, leading to end organ damage. Antibodies against acetylcholine receptors are present in high abundance in patients with myasthenia gravis, however, antibodies directed against muscle-specific kinase (MuSK), low-density lipoprotein receptor-associated protein (Lrp4) or agrin can also be found and are known to interfere with cholinergic transmission between nerve endings and muscle fibers. Disruption of normal communication between nerves and muscles, results in variable weakness of voluntary muscle groups (Berrih-Aknin and Le Panse 2014).

Biomarkers currently used in MG belong primarily to the diagnostic category. Detection of antibodies against the acetylcholine receptor (AChR) or muscle-specific kinase (MuSK) is highly specific for confirming the diagnosis of MG. However, available data indicate that approximately 15% of patients with generalized MG and 50% of patients with ocular type remain seronegative for both AChR and MuSK. Additionally, levels of clinically used MG biomarkers do not correlate with disease severity or clinical response (Berrih-Aknin et al. 2014). Due to the fact that the diagnostic methods used are not conclusive and satisfactory for MG, especially in the early stages, it is necessary to develop reliable tools to help diagnose MG as early as possible (Kaminski et al. 2012).

Metabolomics offers opportunities to identify potential markers associated with disease progression and response to treatment of complex neurological diseases (Hassan-Smith et al. 2012). Interest in amino acids (AA) as potential biomarkers of MG results from their role in the autoimmunity process and disorders in the construction of muscle proteins. Current data suggest that immunoregulatory cells may play significant role in the pathogenesis of MG. Amino acids are important in T cell functioning and their disrupted metabolism may be linked to autoimmunity and related pathology. It appears that their central role in the control of the immune response is underwritten by being indispensable for the generation of building blocks needed for cell and related processes.

Metabolic profiling using amino acid detection has many potential applications. Scientists are focused on their use in screening, diagnostics and treatment monitoring. Recent studies indicate the amino acid profile as a potential marker for the detection of many groups of diseases, such as neurodegenerative diseases (Socha et al. 2019) (Alzheimer's disease (AD) (Corso et al. 2017) Parkinson's (Figura et al. 2018), autoimmune diseases (e.g., Lupus (Kono et al. 2021)) and cancer (Lieu et al. 2020).

The aim of the study is to use HPLC with mass spectrometry detection in conjunction with multivariate statistical analyses to determine changes in the amino acid metabolic profiles of MG patients compared to controls. In addition, the effect of treatment regimens, clinical status and MG forms on the observed changes in amino acid metabolic profiles were also investigated.

## Materials and methods

### Chemicals and reagents

Amino acids standards, internal standard of homoarginine (HARG), methionine-d3 (Met-d3) and homophenylalanine (HPHE) and the derivatisation reagents were included in the EZ:faast^(^™^)^ LC–MS Free Amino Acid kit (Phenomenex, Torrance, CA, USA). HPLC grade methanol was obtained from Merck (Darmstadt, Germany). Ammonium acetate and formic acid of analytical grade were purchased from Sigma-Aldrich Co. (St. Louis, Mo, USA) with purity greater than 99%. Water was deionized and purified using an Milli-Q system (Millipore, Bedford, MA, USA) and used to prepare all aqueous solutions.

### Instrumentation and conditions

The HPLC system consisted of a binary Nexera XR LC-20 AD pump (Shimadzu, Kyoto, Japan) and a Nexera XR SIL-20AC autosampler (Shimadzu, Kyoto, Japan). The chromatography was performed with an EZ: faast^(^™^)^ LC–MS Free Amino Acid kit column (250 × 3.0 mm, 4 µm). A binary gradient elution was carried out with mobile phases A (10 mM ammonium formate in water) and B (10 mM ammonium formate in methanol). The mobile phase flow was 0.25 mL/min and took place in a gradient system: 68% B–83% B in 13 min. The injection volume was 1 µL.

Triple quadrupole tandem mass spectrometric detection was conducted on an LCMS-8045 Mass Spectrometer (Shimadzu, Kyoto, Japan). Electrospray ionization (ESI) mass spectrometry was performed in the positive mode. Multiple reacting monitoring was used for quantification by monitoring ion transition of amino acids. Selected mass spectrometry and chromatography parameters, are summarized in Table 1. LabSolutions software was used for instrument control and quantification. The EZ:faast^(^™^)^ LC–MS Free Amino Acid kit was utilized for serum sample preparation.

**Table 1:** Amino acids, AA abbreviated name, retention time (min.), transitions chosen for each compound

Compound name	Abbreviate name	*t*_R_ (min)	Quantification transition	Confirmation transition
Serine	SER	3.885	233.9 > 146.00	233.9 > 174.00 233.9 > 216.00
Glutamine	GLN	3.468	275.1 > 172.00	275.1 > 84.00 275.1 > 215
Arginine	ARG	3.557	303.1 > 69.00	303.1 > 156.1 303.1 > 286.1
Citrulline	CIT	3.549	304.1 > 156.00	304.1 > 13.00 304.1 > 287.10
Homoarginine	HARG (IS)	3.753	317.10 > 84.00	317.10 > 128.00 317.10 > 170.00
Asparagine	ASN	3.985	243.10 > 157.00	243.10 > 115.00 243.10 > 201.00
1-Methyl-L-histidine	1MHIS	4.201	298.1 > 96.00	298.1 > 196.00
3-Methyl-L-histidine	3MHIS	4.235	298.1 > 210.00	298.1 > 256.00
4-Hydroxyproline	HYP	4.183	260.1 > 172.1	260.1 > 157.50 260.1 > 200.10
Glycine	GLY	4.423	203.9 > 144.00	203.9 > 118.1 203.9 > 162.0
Threonine	THR	4.482	248.10 > 160.00	248.10 > 188.00 248.10 > 230.10
Alanine	ALA	5.347	218.00 > 130.00	218.00 > 158.10 218.00 > 88.00
Gamma-aminobutyric acid	GABA	5.739	232.00 > 172.00	232.00 > 130.00
Sarcosine	SAR	5.948	217.90 > 88.00	217.90 > 158.00 217.90 > 116.00
Beta-amino isobutyric Acid	BAIB	6.202	232.00 > 172.00	232.00 > 130.00
*α*-Aminobutyric acid	ABA	6.567	232.00 > 172.00	232.00 > 130.00 232.00 > 134.10
Ornithine	ORN	6.795	347.00 > 287.00	347.00 > 156.00 347.00 > 227.10
Methionine	MET	7.085	227.9 > 190.10	227.9 > 218.00 227.9 > 142.00
Methionine-d3	Met-d3 (IS)	7.030	281.1 > 193.00	281.1 > 221,000 281.1 > 142,00
Proline	PRO	7.130	244.00 > 156.00	244.00 > 114.00 244.00 > 184.00
Lysine	LYS	7.743	361.00 > 301.10	361.00 > 170.10
Aspartic acid	ASP	7.753	304.00 > 216.10	304.00 > 130.00 304.00 > 244.00
Histidine	HIS	7.826	370.10 > 196.00	370.10 > 110.10 370.10 > 284.10
Valine	VAL	8.116	246.00 > 158.00	246.00 > 116.00 246.00 > 186.00
Glutamic acid	GLU	8.223	317.50 > 172.10	317.50 > 258.00 317.50 > 230.30
Tryptophan	TRP	8.468	333.10 > 245.10	333.10 > 273.00 333.10 > 230.00
*α*-Aminoadipic acid	AAA	9.202	332.00 > 244.10	332.00 > 185.50 332.00 > 272.10
Leucine	LEU	9.593	260.00 > 172.10	–
Phenylalanine	PHE	9.679	294.10 > 206.10	294.10 > 120.10
Isoleucine	ILE	9.997	260.00 > 172.10	260.00 > 130.00 260.00 > 74.10
Homophenylalanine	HPHE (IS)	11.224	308.00 > 220.00	308.00 > 117.00 308.00 > 104.10
Cystine	C–C	11.402	497.00 > 248.00	497.00 > 437.00 497.00 > 306.00
Tyrosine	TYR	11.999	395.90 > 136.00	395.90 > 308.00 395.90 > 336.00

### Statistical analysis

The Kruskal–Wallis test was used to assess differences between continuous variables distribution (age, amino acids concentration) in relation to MG subtypes. Post-hoc analysis was further used to explore differences between means while controlling the family error rate. Considering non-normally distributed data, differences between two groups were evaluated using *U*-Mann–Whitney test statistics. Bonferroni correction was used to adjust for multiple testing. Pearson’s correlation was used to study the linear relationship between AAs. All analyses and figures were done in Python [Van Rossum, G., & Drake, F. (2009). Python 3 Reference Manual. Scotts Valley, CA: CreateSpace].

### Subjects and serum samples

A total of 81 participants were recruited from the Sanitas–Neurology Outpatient Clinic, Bydgoszcz, Poland. They were divided into two groups: 53 healthy volunteers with no history of autoimmune diseases (mean age ± SD: 58.38 ± 12.78; 30 men, 23 women) and 28 patients with generalized or ocular subtype of myasthenia gravis (mean age ± SD: 48.92 ± 12.61; 3 men, 25 women). Table 2 shows the clinical and demographic characteristics of the study group.

**Table 2:** Characterization of myasthenia gravis patients (*n* = 28) in relation to selected covariates (age, sex, drug treatment, presence/absence of thymus, duration of disease, expression of AchR receptor)

Myasthenia gravis (*n* = 28)			
**Age**	48.92 (± 12.61)	**Duration (mean)**	7.85 (± 6.53)
**Sex**		**Disease subtype**	
Female	25 (89.3%)	Generalized	25 (89.3%)
Male	3 (10.7%)	Ocular	3 (10.7%)
**Drug**		**MGFA**	
Mestinon	7 (25%)	I	3 (10.7%)
Mestinon, steroid	8 (28.5%)	II A	8 (28.5%)
Mestinon, steroid, azathioprine	8 (28.5%)	III A	12 (42.8%)
Mestinon, steroid, cyclophosphamide	1 (3.5%)	III B	1 (3.5%)
Mestinon, steroid, methotrexate	1 (3.5%)	IV A	3 (10.7%)
Mestinon, steroid, Mycophenolate mofetil	3 (10.7%)	IVA	1 (3.5%)
**Thymus presence**		**Thymectomy**	
No	10 (35.7%)	No	10 (35.7%)
Yes	18 (64.3%)	Yes	18 (64.3%)
**AchR**		**Thymoma**	
No	7 (25%)	No	7 (25%)
Yes	21 (75%)	Yes	1 (3.5%)

This study was conducted in accordance with all internationally approved human testing guidelines and in accordance with the Declaration of Helsinki. The Ethics Committee of the Nicolaus Copernicus University in Toruń and Collegium Medicum in Bydgoszcz approved this study (consent number: KB 135/2019).

Antecubital whole-blood samples were drawn from a peripheral vein in the morning hours (always between 7 and 8 a.m.). Overnight fasting and 15 min of rest before the blood test were obligatory. Serum from the blood after clotting was separated out and, after appropriate preparation and centrifuging, was frozen at − 80 °C until analysis was performed.

## Results

### Comparison of amino acid concentration in myasthenia gravis patients (*n* = 28) versus control group (*n* = 53)

**Fig. 1:**
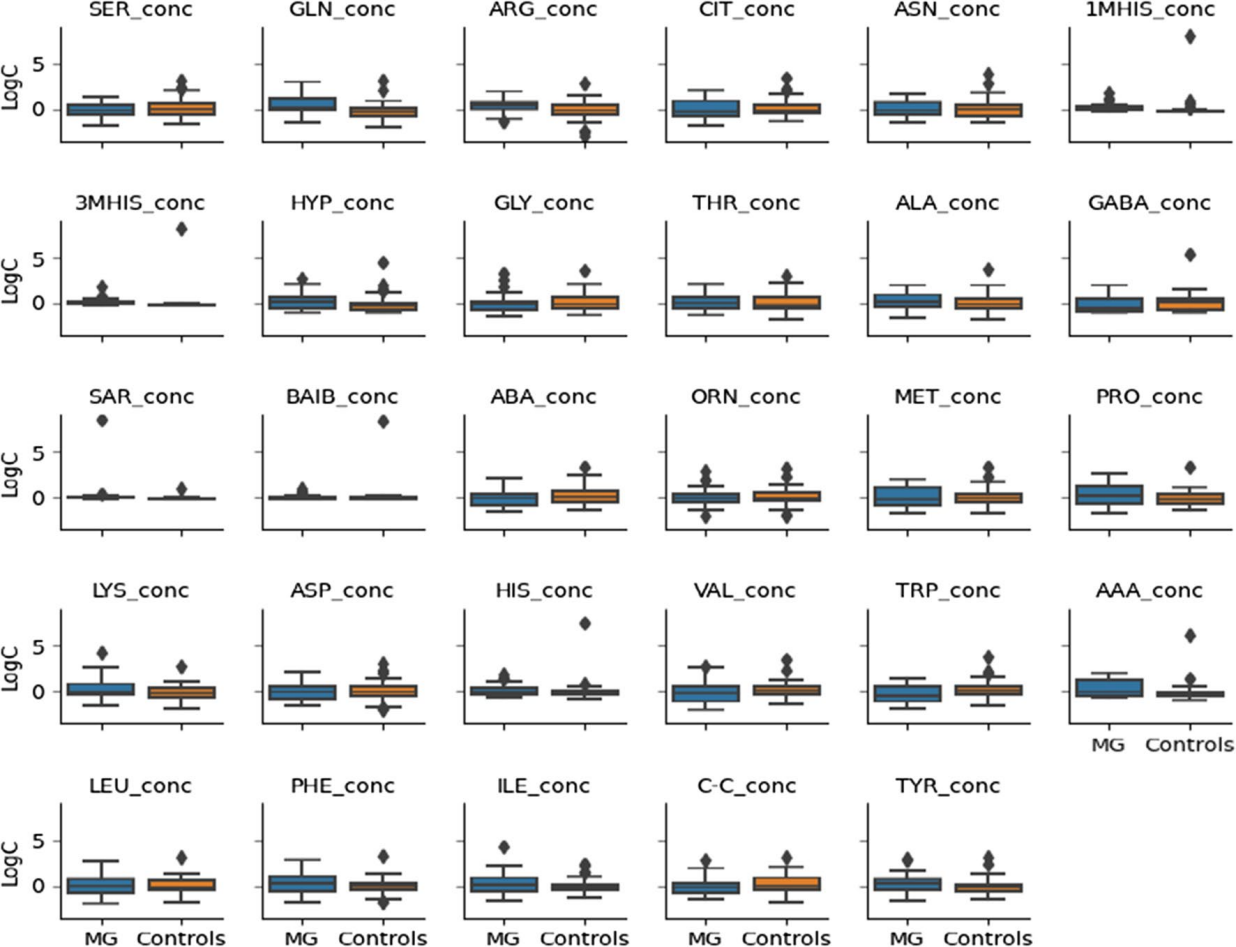
Centered and standardized 29 amino acids concentrations between myasthenia gravis (MG) patients (*n* = 28) and healthy individuals (controls (*n* = 53) (orange) represented as boxplots. Black dots denote outlying observations

To maintain the same scale for each AA and for data analysis purposes, the raw values were centered and standardized. Figure 1 presents a visualization of the centered and standardized data for each AA for the two MG patients and controls.

**Fig. 2:**
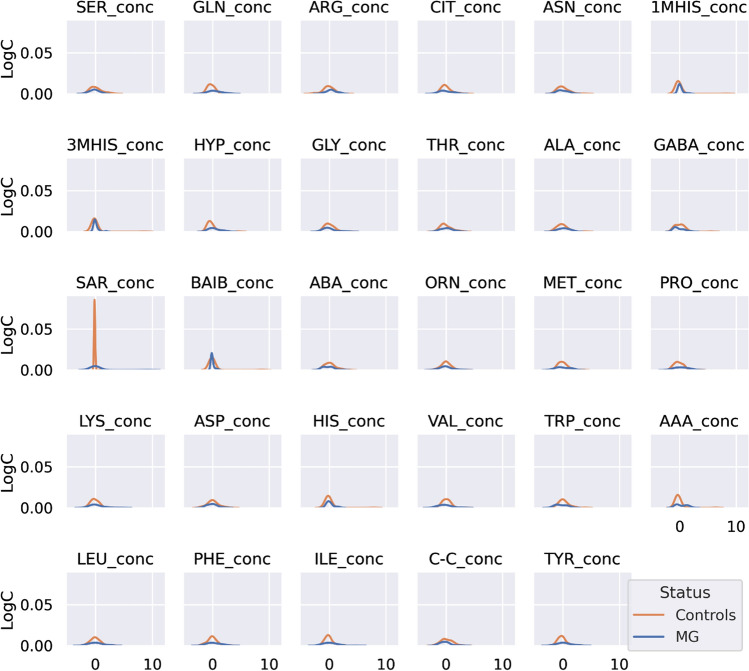
Density plot of the centered and standardized 29 amino acids concentrations between myasthenia gravis patients (*n* = 28) and controls (*n* = 53) (orange)

From visual inspection one can observe slight difference in AA concentration between both groups. To investigate whether AA distributions were homogeneous and whether the AA concentration data originated from two or more different overlapping distributions (e.g., groups), the distribution of each AA in the control (*n* = 53) and case (*n* = 28) groups was provided (Fig. 2).

Distribution of each AA between both groups was different (*α* = 0.05) for GLN (*p* = 0.011), ARG (*p* = 0.031), 1MHIS (*p* = 0.000001), 3MHIS (*p* = 0.0001), HYP (*p* = 0.012), SAR (*p* = 0.02).

**Fig. 3:**
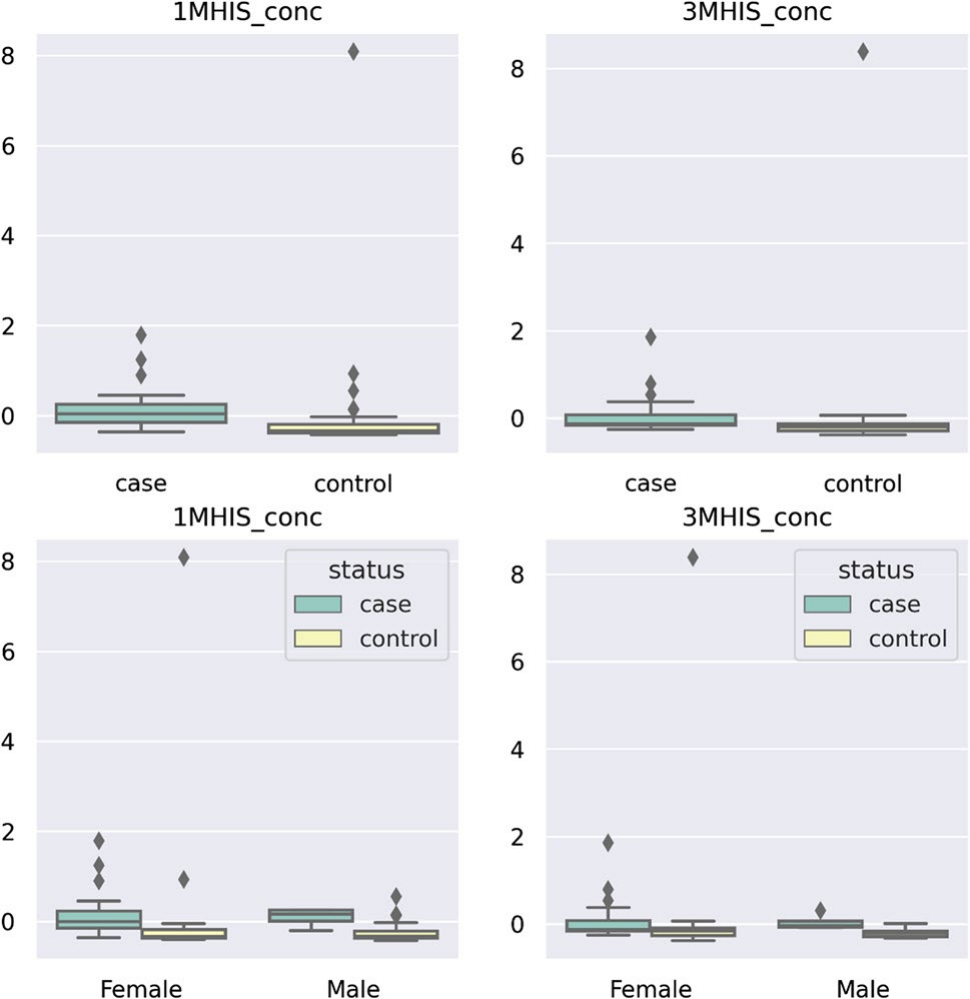
Centered and standardized concentration of 1MHIS and 3MHIS between myasthenia cases (*n* = 28) and controls (*n* = 53). Black dots represent outlying observations

Simultaneous testing of many hypotheses (so-called families of hypotheses) entails the danger of increasing the *α* error. Hence, when correcting for multiple testing (Bonferroni correction) (corrected *p* value = 0.0017), concentration of 1MHIS and 3MHIS remained significant. Figure 3 presents the cantered and standardized concentration of 1MHIS and 3MHIS between myasthenia cases and controls, also taking into account the division of the studied groups by gender.

**Fig. 4:**
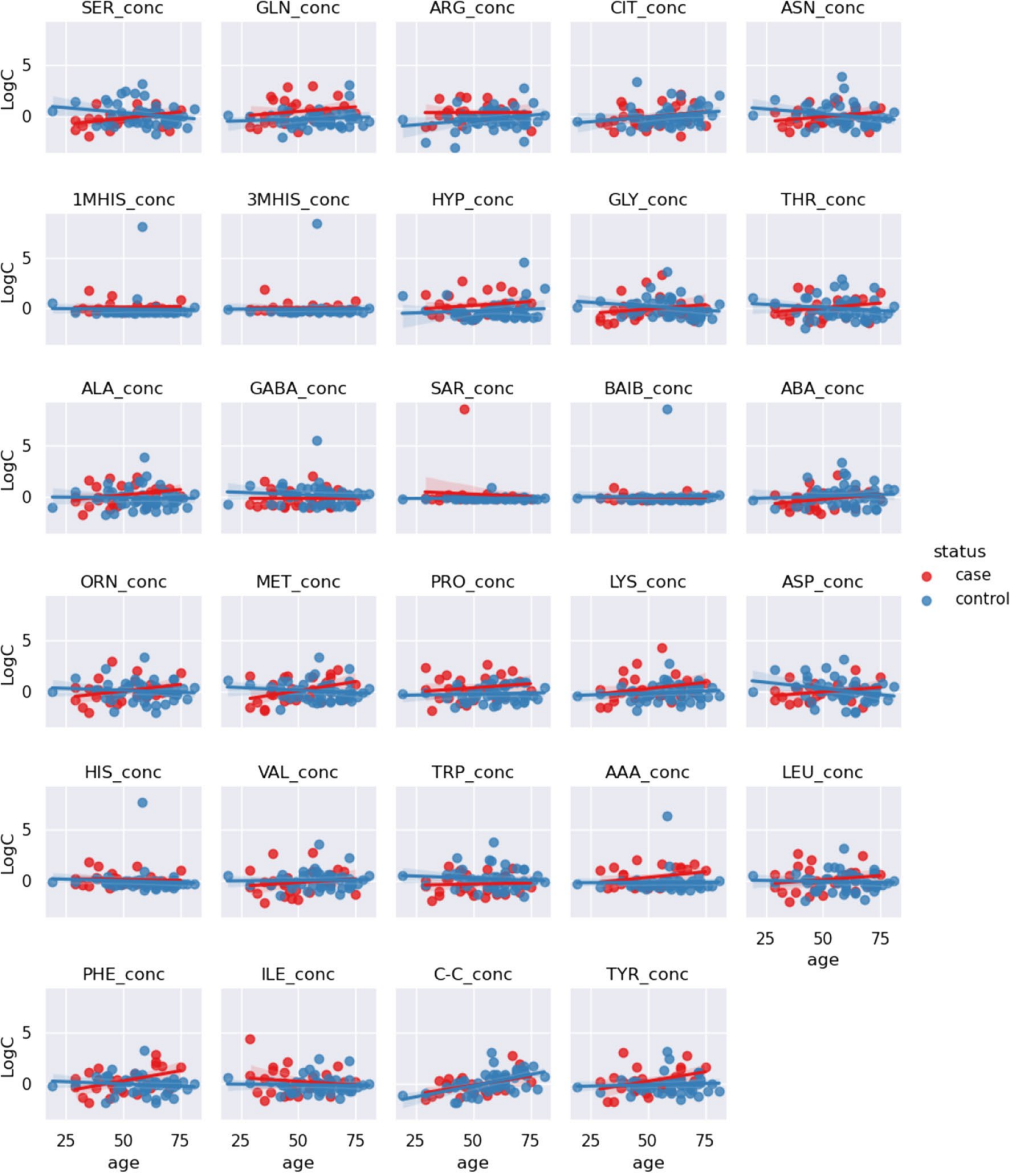
Centered and standardized 29 amino acids concentrations between two groups – myasthenia gravis (*n* = 28) and controls (*n* = 53) (blue). Red and blue dots denote cases and controls. Red and blue lines are regression lines representing a trend of amino acids concentration with age. Shaded line around regression lines denotes 95% confidence interval

In the next step, we checked if there is a trend associated with age in terms of AA concentration between myasthenia cases and controls (Fig. 4). From visual inspection, in terms of patients, there was no clear linear trend (positive or negative) with increasing age (red line).

### Analysis of changes in AA concentrations in the context of the duration of myasthenia gravis

**Fig. 5:**
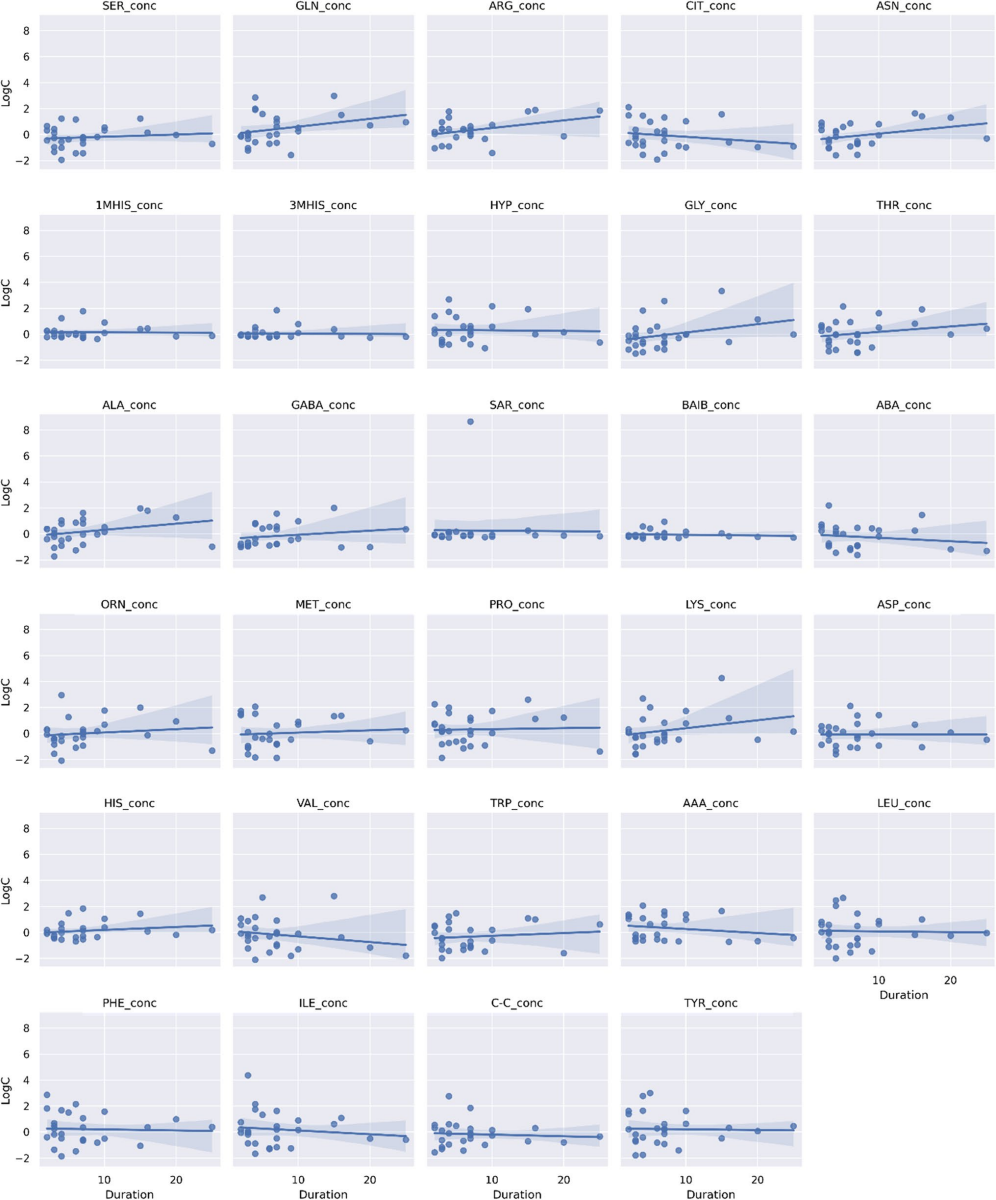
Centered and standardized amino acids concentration in myasthenia patients in relation to disease duration. Blue line represents linear regression line

Figure 5 presented 29 centered and standardized AA concentrations signals in a function of myasthenia duration to check for any trend in (e.g., higher concentration of AA along with myasthenia duration or lower concentration with myasthenia duration). Visual inspection shows a slightly positive trend in the concentration of GLN, ARG, ASN in a function of duration. Slightly negative trend is observed for CIT concentration. One should highlight the wide confidence intervals around regression line (due to low sample size and wide range of measurements).

### Comparison of AA concentrations by subtype of myasthenia gravis

**Fig. 6:**
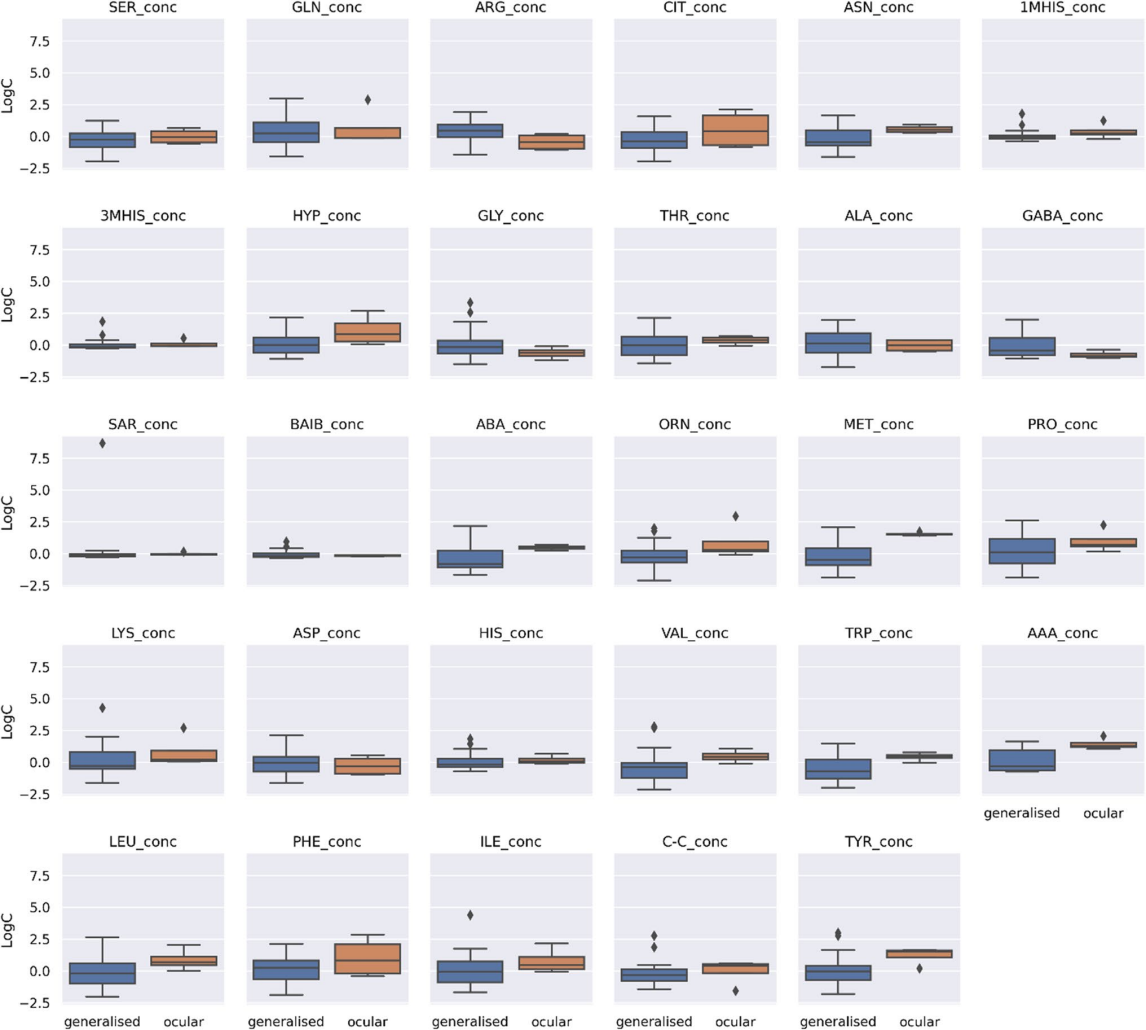
Centered and standardized concentration of 29 amino acids in myasthenia gravis patients according to subtype. i.e., ocular (*n* = 3) and generalized (*n* = 25). Black dots represent outlying observations

**Fig. 7:**
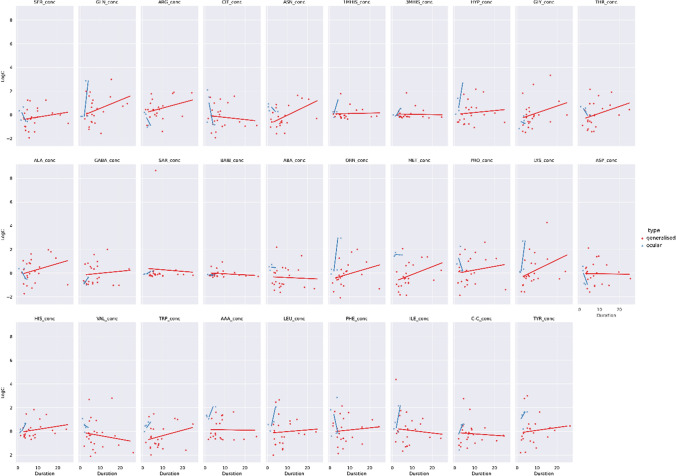
Centered and standardized AA concentration in myasthenia patients subtype in relation to disease duration

Concentrations of 29 AA were plotted according to the subtype of myasthenia gravis (generalized vs. ocular) and it was checked whether there are differences in AA concentrations depending on the subtype of myasthenia gravis. Figure 6 presents a visualization of the cantered and standardized data for each AA for these two groups. It was found that concentration of ABA (*p* = 0.025) and AAA (*p* = 0.024) were significantly different between generalized (*n* = 25) and ocular (*n* = 3) group. After multiple testing correction both AA were non-significant. Next, the trend of AA concentrations was checked with the duration of myasthenia gravis according to subtype of myasthenia gravis (Fig. 7). Different trend directions can be observed for each disease subtype.

### Evaluation of differences in AA concentrations between myasthenia gravis classification schemes according to MGFA

**Fig. 8:**
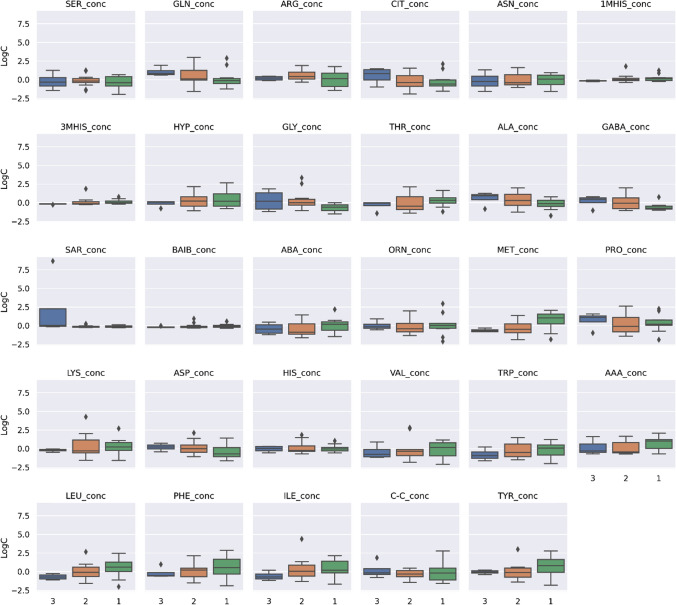
Centered and standardized 29 amino acids’ concentrations of myasthenia patients (*n* = 28) according to MGFA classification. On the x axis, 3 categories are depicted: ‘1’, ‘2’, ‘3’. The ‘1’ denotes **I + II A**, ‘2’ denotes **III A + III B** and ‘3’ is **IV A**. Black dots denote outlying observations

The Myasthenia Gravis Foundation of America (MGFA) clinical classification divides MG into main classes and several subclasses. It is designed to identify subgroups of patients with MG who share distinct clinical features or severity of disease that may indicate different prognoses or responses to therapy. The differences in AA concentrations between the MGFA classification schemes were checked (Kruskal–Wallis test). Patients with myasthenia gravis were divided into 3 groups: 1. I + II A (*n* = 11); 2. IIIA + IIIB (*n* = 13); 3.IVA (*n* = 4). Figure 8 presents a visualization of centered and standardized data for each AA for this three groups. From visual inspection a slight difference in AA concentration can be observed between the MGFA groups. Post-hoc analysis revealed no such differences.

### Evaluation of differences in AA concentrations, taking into account the treatment regimens of patients

**Fig. 9:**
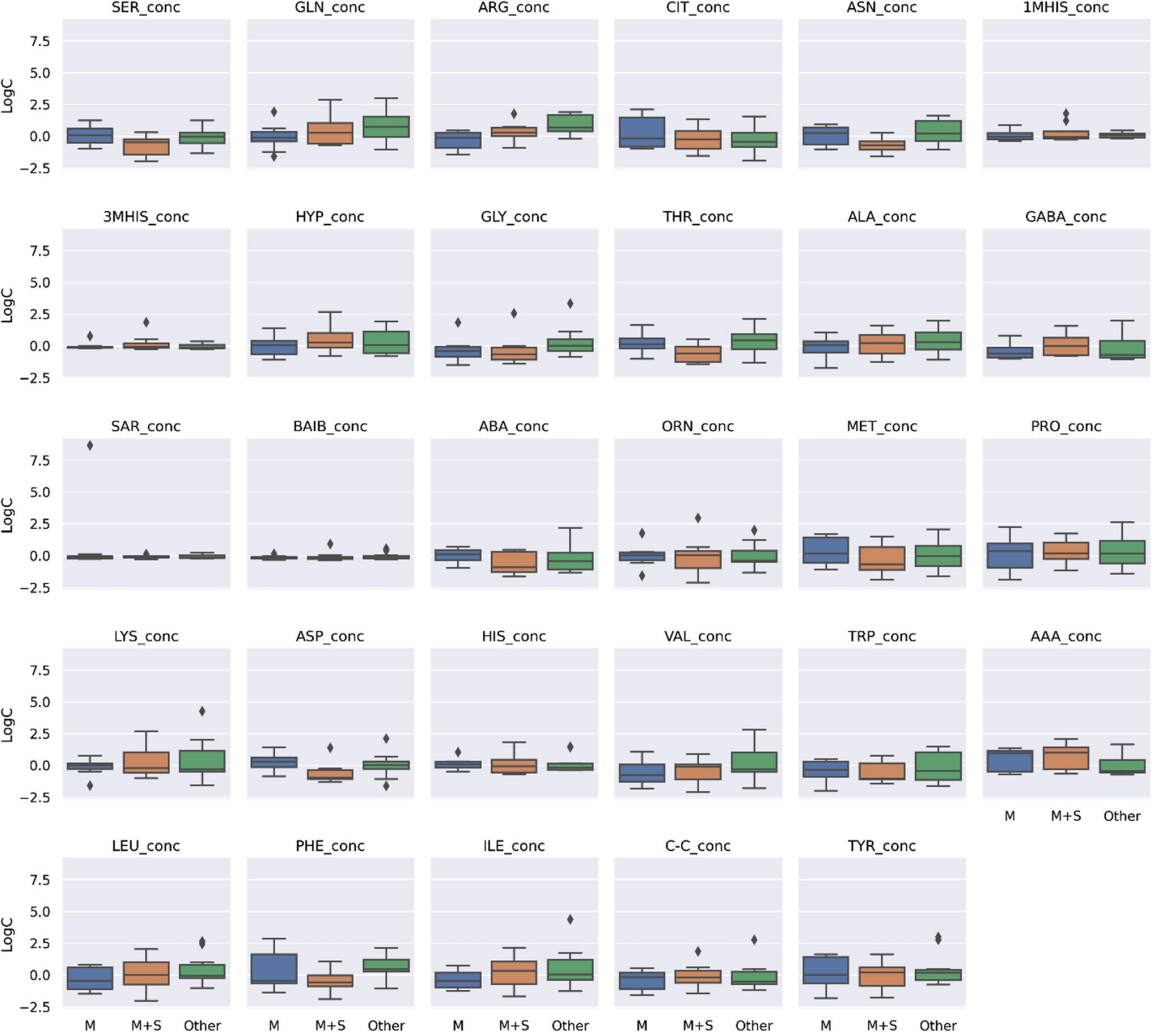
Centered and standardized 29 amino acids concentrations of myasthenia patients (*n* = 28) according to treatment scheme. On the x axis, 3 categories are depicted: ‘M’, ‘M + S’, ‘Other’. ‘M’ denotes mestinon treatment, ‘M + S’ denotes mestinon + steroid treatment and ‘Other’ is the treatment composed of mestinon, steroid and other drugs such as azathioprine, mycophenolate mofetil, cyclophosphamide**,** methotrexate. Black dots denote outlying observations

We also investigated the relationship between AA concentrations and treatment schema distinguishing between 3 treatment schemes: (i) mestinon treatment (M), (ii) mestinon + steroid treatment (M + S) and (iii) treatment composed of mestinon, steroid and other immunosuppressive drugs such as azathioprine, mycophenolate mofetil, cyclophosphamide, methotrexate (Other). Figure 8 presents a visualization of the centered and standardized data for each AA for these three groups. Post-hoc analysis revealed differences between mestinon treatment (M) and treatment composed of mestinon, steroid and other drugs such as azathioprine, mycophenolate mofetil, cyclophosphamide, methotrexate (Other) for ARG (*p* = 0.034), and also between mestinon + steroid (M + S) and treatment composed of mestinon, steroid and other drugs such as azathioprine, mycophenolate mofetil, cyclophosphamide, methotrexate (Other) for ASN (*p* = 0.044) (Fig. 9).

### Comparison of AA concentrations depending on the presence of thymus, thymectomy, AchR, presence of thymoma

AA concentrations were also compared according to the presence of thymus, thymectomy, AchR, presence of thymoma. Based on the statistics of the Mann–Whitney U test, there were no differences in AA concentrations depending on the presence or absence of thymus, thymectomy or AchR.

### Evaluation of changes in amino acid correlations between the study group and the control group

**Fig. 10:**
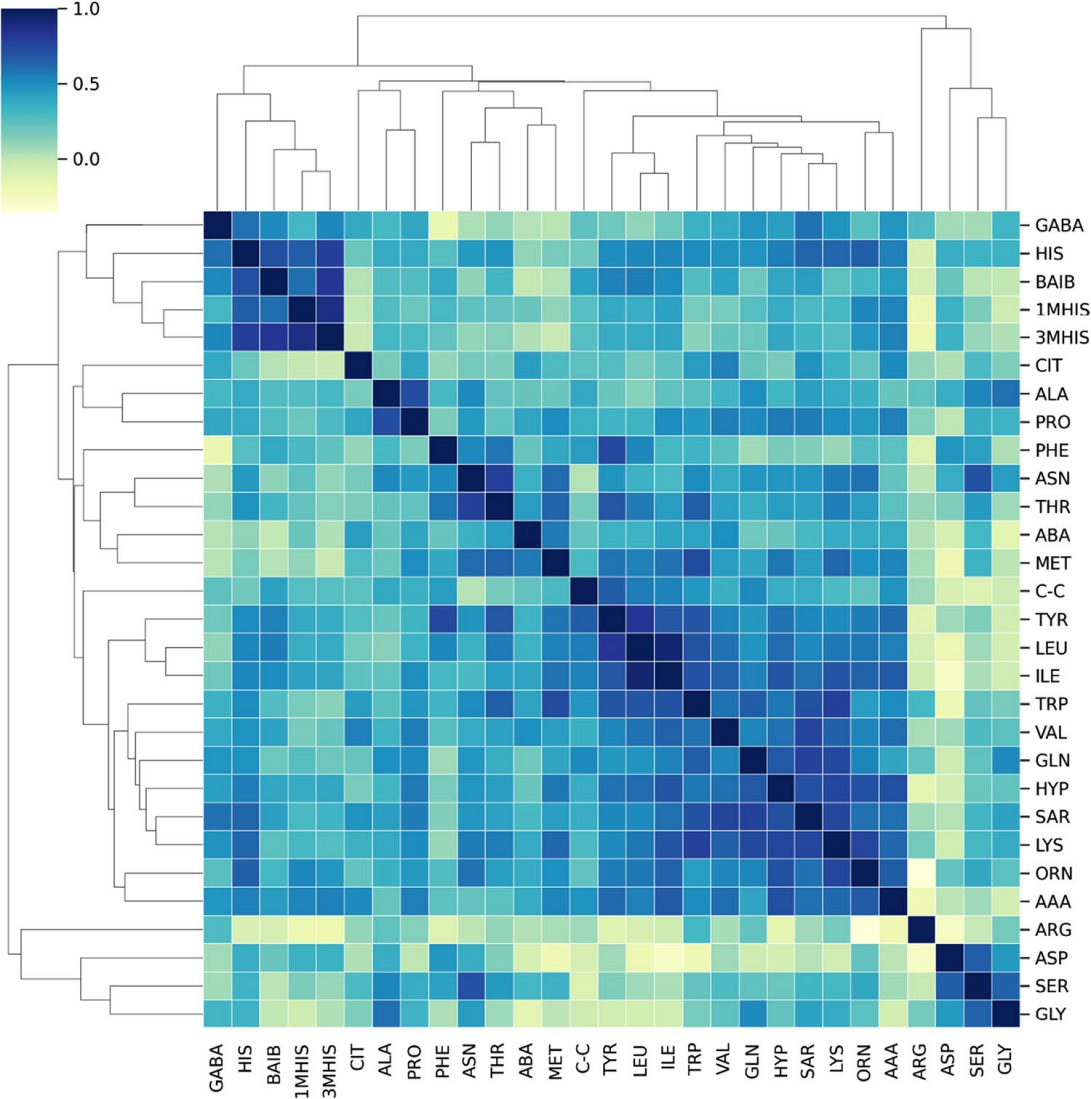
Correlation matrix demonstrating the degree of linear relationship between amino acids in the group of myasthenia patients. The darker the color the higher coefficient value between two amino acids

**Fig. 11:**
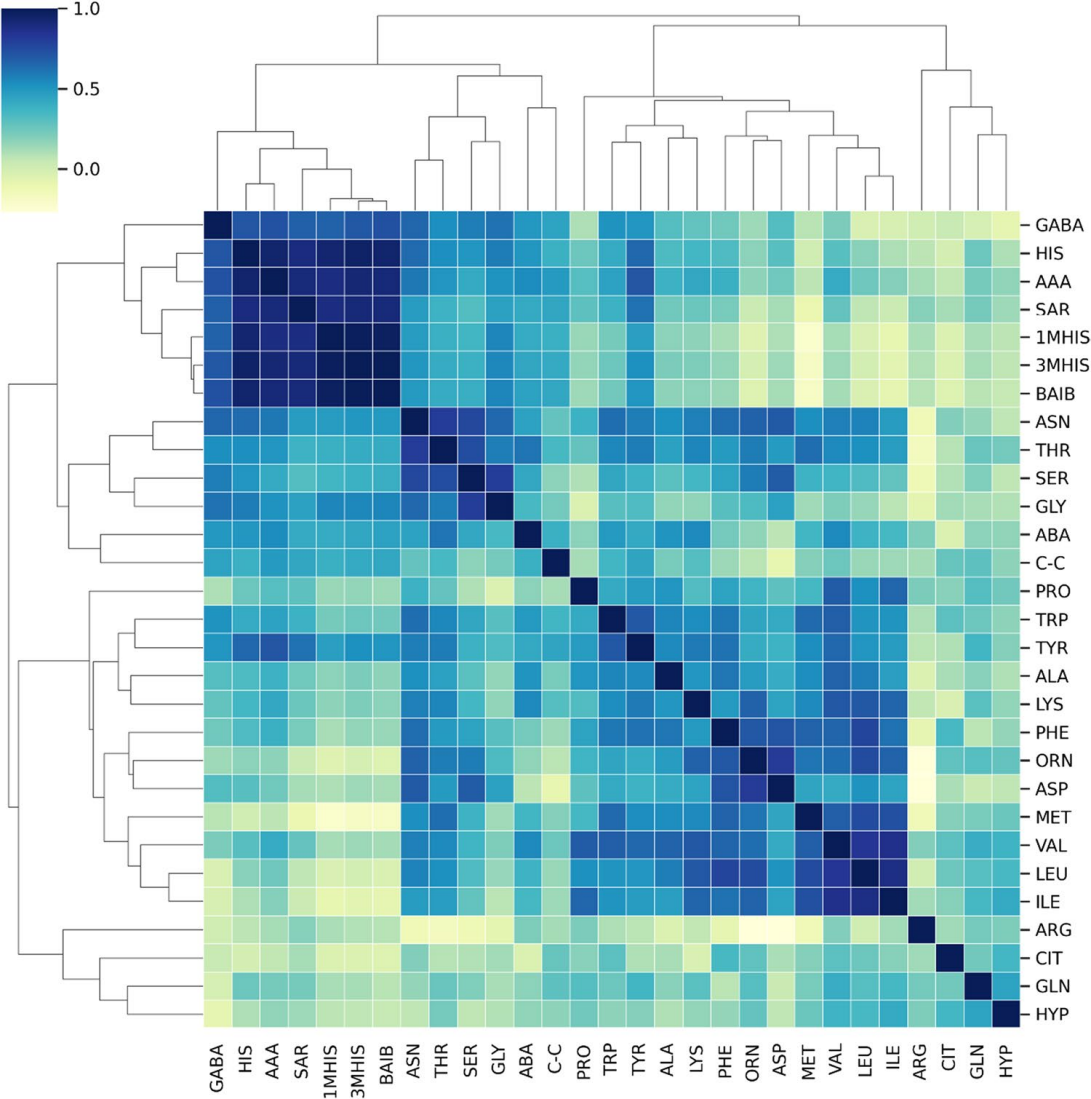
Correlation matrix demonstrating the degree of linear relationship between amino acids in the controls. The darker the color the higher coefficient value between two amino acids

To further investigate the nature of amino acids, the correlations between amino acids were checked. Since the distribution for each AA resembles a normal distribution and the AAs follow a similar pathway, a linear relationship between them can be expected. Therefore, the Pearson correlation, which measures the linear relationship between the variables, was used. The correlation matrix showing the degree of linear relationship between AA in patients with myasthenia gravis and the control group is presented in Figs. 10 and 11, respectively. As observed in the figure presenting myasthenia gravis patients, a vast majority of correlations in positive. A bunch of AA are positively correlated with other AA (*R*^2^ > 0.8, dark blue) and essentially they carry the same information. The heatmap for patients seems to present weaker positive correlations between AA.

## Discussion

Metabolomics (Kaddurah-Daouk and Krishnan 2009) is a rapidly developing field that aims to identify and quantify the concentration changes of all the metabolites (i.e., the metabolome) in a given biofluid from a subject and support targeting and developing therapeutics. The application of metabolomics towards understanding the manifestation and progression of complex neurological diseases represents a powerful means to identify the earliest markers associated with disease progression and treatment response (Kohler et al. 2016). Metabolomics strategies have been divided into two distinct approaches, untargeted and targeted metabolomics. Untargeted metabolomics is the comprehensive analysis of all the measurable analytes in a sample, including chemical unknowns. Targeted metabolite profiling sets out to gain a greater understanding of a biological pathway or class by quantifying a distinct selection of metabolites (Iman et al. 2022).

Myasthenia gravis (MG) is a T cell-mediated autoimmune disease mediated by B cells. This disease is caused by antibodies against the nicotinic acetylcholine receptor or other components of the postsynaptic muscle endplate at the neuromuscular junction (Wu et al. 2020). Diagnostic methods used in MG often do not give satisfactory results, especially in the early stages of the disease. Therefore, the need to develop reliable tools to help diagnose MG as early as possible is emphasized. New diagnostic methods should be less invasive with high sensitivity and specificity.

Results of the previous studies focusing on non-targeted metabolic analysis in MG patients allowed to confirm the differences in metabolic profiles between the study and control groups. In the work of Lu et al., an approach of non-targeted serum metabolic analysis using LC–MS was used. The obtained results allowed to clearly distinguish healthy individuals from patients with myasthenia gravis based on their global metabolic profiles. In addition, different changes in metabolic profiles were observed between early-stage and late-stage MG patients (Lu et al. 2012). The study by Sengupta et al. analyzed the serum metabolic profile collected from MG patients before and after 12 weeks of prednisone treatment during a clinical trial. The aim of this study was to identify metabolites that may respond to treatment and that could be evaluated in future studies as potential biomarkers of efficacy or side effects. Untargeted metabolic profiling of serum showed a clear distinction between pre- and post- treatment groups. Disturbances in the metabolism of amino acids, carbohydrates, vitamins and lipids have been observed. (Sengupta et al. 2014). Blackmore et al. used a new approach to accurately identify unique serum metabolomic biomarkers that distinguish seropositive MG patients from the reference autoimmune disease, i.e., rheumatoid arthritis (RA) and healthy controls. The identified patterns of serum biomarkers differed statistically significantly between the study groups (Blackmore et al. 2019).

Interest in amino acids as potential biomarkers of MG results from their role in the autoimmunity process and disorders in the construction of muscle proteins. MG is a T cell-dependent, B-cell mediated autoimmune disease (Wu et al. 2020). T cell metabolism is central to cell proliferation, survival, differentiation, and aberrations have been linked to the pathophysiology of systemic autoimmune diseases (Kono et al. 2021). Current data suggest that immunoregulatory cells may play significant roles in the pathogenesis of MG (Wu et al. 2020).

Amino acids are important in T cell function, and abnormal metabolism can be linked to autoimmunity and related pathology. Their main role in controlling the immune response appears to be that they are essential for generating the building blocks needed for cell proliferation, producing energy by controlling metabolic pathways, controlling epigenetic pathways, producing phospholipids, and controlling oxidative stress (Kono et al. 2021).

The result of our study indicate that the serum level of amino acids changed significantly in patients with myasthenia gravis in relation to control group. In particular we observed differences in concentration for GLN (*p* = 0.0110, Arg (*p* = 0.031), 1MHIS (*p* = 0.000001), 3MHIS (*p* = 0.0001), HYP (*p* = 0.012), SAR (*p* = 0.02) at *p* < 0.05.

The observed differences in glutamine concentrations in MG patients compared to controls may be due to changes in muscle tissue as well as the activity of the immune system. Glutamine is the most abundant and versatile amino acid in the body. The availability and metabolism of glutamine in the body are directly related to i.e., skeletal muscle tissue. Skeletal muscle is quantitatively the most important site for glutamine accumulation, synthesis and release. Skeletal muscles, therefore, play a fundamental role in glutamine metabolism, as it is one of the most abundant tissues in the human body (Cruzat et al. 2018). Glutamine metabolism also plays an important role in energy production in proliferating cells, including T cells (Kono et al. 2021). It is estimated that the rate of consumption of glutamine by immune cells is similar or greater than that of glucose. In vitro and in vivo studies have shown that glutamine is an essential nutrient for lymphocyte proliferation and cytokine production, phagocytic and secretory activity of macrophages. The enzymes involved in glutaminolysis have been extensively studied. Particularly noteworthy, in the context of autoimmunity, is the role of glutaminolysis in the production of pro-inflammatory effector T lymphocytes Th1 and Th17. Glutaminase, responsible for converting glutamine to glutamate, promotes Th17 cells through various mechanisms (Kono et al. 2018) (Kato and Perl 2014). Transcription factor-induced cAMP early repressor (ICER)/cAMP response element modulator (CREM) is involved in glutaminase expression. Previous studies have shown that it is also overexpressed in T lymphocytes in both SLE patients and lupus-prone MRL/lpr mice (Tenbrock et al. 2002; Yoshida et al. 2016).

Arginine, the concentration of which determined in our study allows for the differentiation of the study group and the control group, is involved in the immune response. L-Arg is an integral component of proteins. Arginine, by participating in the synthesis of non-protein compounds such as nitric oxide or polyamines, plays an important role in vasodilation, calcium release, neurotransmission and immune response. ARG-dependent circuitry has been hypothesized to play a role in physiological and pathological immune responses, but the molecular details have not been fully defined (Shearer et al. 2018). Scientific evidence supports the assumption of the important role of NO-dependent mechanisms in various autoimmune diseases or chronic inflammatory conditions. The release of large amounts of NO, a powerful pro-inflammatory agent, by activating NOS2, can exacerbate inflammation. Studies of autoimmune demyelinating diseases, such as multiple sclerosis, confirm the presence of oxygen and nitrogen free radicals. In fact, analysis of cerebrospinal fluid (CSF) from MS patients shows increased nitrate and nitrite levels compared to healthy controls (Sellebjerg et al. 2002).

In the subsequent stages of statistical analysis, the Bonferroni correction was used, which allowed the identification of two amino acids derived from histidine (1MH and 3MH), which most significantly differentiated the studied groups of patients with MG and the control group in terms of statistical significance.

L-Histidine (HIS) is an essential amino acid (EAA) with a wide range of biochemical and physiological properties. 3-Methyl histidine (3MH) is formed as a result of post-translational modification of histidine residues. It has been identified in both actin and myosin as part of myofibrillar proteins. The presence of free 3MH in the body is the result of i.a. breakdown of these proteins. Due to the fact that 3MH is not metabolized or reused for protein synthesis, it has been proposed as a non-invasive marker of myofibrillar protein catabolism (Biotcch Bioc et al. 1996; Sjijlin et al. 1987). An increase in the rate of myofibrillar protein breakdown has been observed in a variety of muscle-wasting pathologies. In these situations, progressive loss of muscle protein occurs when the rate of protein degradation exceeds the rate of protein synthesis. Such a clinical presentation was observed e.g., in patients with Duchenne, Becker or myasthenia gravis (Warnes et al. 1981)]. It is recognized that 1-MH is not formed in humans and its presence results from the metabolism of anserine dipeptide obtained from food. 1-MH represents a potentially useful marker of meat consumption (Holeček 2020; Myint et al. 2000). The use of the Pearson correlation, which measures a linear relationship between variables, also allowed us to observe some differences between the group of patients with MG and the control group. Although mainly positive relationships between AAs were observed, these linear relationships seem to be slightly weaker in the control group compared to the study group.

In the present study, the dominant group of patients with myasthenia gravis was of generalized type. Despite the fact that the groups with the ocular and generalized types are not balanced, the obtained results indicate the possibility of differentiating the types of myasthenia gravis using amino acid profiles. It was found that concentration of ABA (*p* = 0.025) and AAA (*p* = 0.024) were significantly different between generalized (*n* = 25) and ocular (*n* = 3) group. After Bonferroni correction for multiple testing both AA were non-significant. We also reported different directions of trend when considering AA concentration according to disease subtypes. This indicates the need for further research with a balanced representation of both forms of MG.

One of the goals of our study was to try to identify amino acids that may respond to treatment and that could be used in future studies as potential biomarkers of treatment effectiveness or side effects. Individualization of drug therapy, as a result of different responses to pharmacological agents, is aimed at minimizing adverse reactions to the drug, while maximizing the therapeutic effect. Post-hoc analysis revealed the differences between mestinon treatment (M) and treatment classified as ‘Other’ composed of mestinon, steroid and other drugs such as azathioprine, mycophenolate mofetil, cyclophosphamide, methotrexate for ARG. The obtained results also allowed for the identification of differences between mestinon + steroid (M + S) treatment and treatment composed of mestinon, steroid and other drugs immunosuppressive such as azathioprine, mycophenolate mofetil, cyclophosphamide, methotrexate for ASN (*p* = 0.044). Myasthenia gravis treatment monitoring study was conducted by Sengupta et al. (2014). The aim of study was to identify metabolites that may be responsive and can be evaluated in future studies as potential biomarkers of efficacy or adverse effects. The obtained results confirmed the possible role of amino acid metabolites (l-phenylalanine, o-acetylserine, N2-acetyl-l-ornithine) in monitoring prednisone treatment.

In the presented study, we could not observe differences in AA concentrations depending on clinical data such as the presence or absence of a thymus, thymectomy, thymoma or antibodies against AchR.

## Conclusions

This study showed that amino acids may be involved in mechanisms underlying myasthenia gravis pathogenesis and may also be considered as potential indicators of certain pathways altered in myasthenia. However, given the multifactorial, heterogeneous and complex nature of this disease, further work with a larger study group is required to correlate the results of previous studies and possibly other autoimmune diseases, to investigate whether the amino acid profile in myasthenia gravis can be considered as characteristic fingerprint of this disease.
